# Calcitonin Gene-Related Peptide and Adrenomedullin Levels During Ictal and Interictal Periods in Patients With Migraine

**DOI:** 10.7759/cureus.37843

**Published:** 2023-04-19

**Authors:** Abdurrahman Neyal, Yasemin Ekmekyapar Fırat, Mustafa B Çekmen, Emine Kılıçparlar Cengiz, Saniye Koç Ada, Ayşe M Neyal

**Affiliations:** 1 Department of Neurology, Gaziantep Islam Science and Technology University School of Medicine, Gaziantep, TUR; 2 Department of Neurology, Sanko University School of Medicine, Gaziantep, TUR; 3 Department of Medical Biochemistry, Istanbul Medeniyet University School of Medicine, Istanbul, TUR; 4 Department of Neurology, Dr. Ersin Arslan Education and Research Hospital, Gaziantep, TUR

**Keywords:** migraine, cgrp, adrenomedullin, ictal period, interictal period

## Abstract

Background

Peptides related to calcitonin gene-related peptide (CGRP) have been suggested to have a role in migraine. Adrenomedullin (AM) might be a candidate molecule because it is related to pain pathways in the peripheral and central nervous systems and uses the same receptors as CGRP.

Methodology

In this study, we examined the serum CGRP and AM levels during unprovoked ictal and interictal periods of 30 migraine patients as well as 25 healthy controls. Another focus of this study was on the association of CGRP and AM levels with clinical features.

Results

Mean serum AM levels were 15.80 pg/mL (11.91-21.43 pg/mL) in the ictal and 15.85 pg/mL (12.25-19.29 pg/mL) in the interictal periods in the migraine group and 13.36 pg/mL (10.84-17.18 pg/mL) in the control group. Mean serum CGRP levels were 2.93 pg/mL (2.45-3.90 pg/mL) in the ictal and 3.25 pg/mL (2.85-4.67 pg/mL) in the interictal periods in the migraine group and 3.03 pg/mL (2.48-3.80 pg/mL) in the control group. There were no statistical differences between ictal and/or interictal AM and CGRP levels (p = 0.558 and p = 0.054, respectively) which were also comparable with the results of the control group (p = 0.230, p = 0.295, p = 0.987, p = 0.139, respectively). Ictal serum CGRP and/or AM levels did not correlate with any of the reported clinical features.

Conclusions

Serum AM and CGRP levels are similar in interictal and unprovoked ictal periods in migraine patients and as well in controls. These results do not indicate that these molecules do not have a role in migraine pathophysiology. Considering the broad mechanisms of action of peptides in the CGRP family, further studies are needed in larger cohorts.

## Introduction

Migraine is a prevalent headache syndrome in the population. It is a neurovascular syndrome that occurs based on genetic predisposition, is triggered by environmental factors, and is characterized by recurrent headache attacks with accompanying symptoms.

Calcitonin, calcitonin gene-related peptide (CGRP), adrenomedullin (AM), and amylin are members of the calcitonin peptide family. All peptides in this family can activate the different receptors of this family [1]. The calcitonin receptor (CTR) and CT receptor-like receptor (CLR) form seven different receptors with different physiological effects and pharmacokinetic behaviors by interacting with the receptor activity-modifying protein (RAMP) [1].

CGRP, a common molecule in pain, may also have a critical role in migraine pathophysiology. Recent studies have indicated that blocking CGRP can be used in migraine treatment [2-4]. Phase 3 studies involving migraine patients revealed that CGRP blockade was significantly more beneficial than placebo [5-7]. Yet, systematic reviews in the following years have reported that CGRP blockade may not have a more substantial effect than the current prophylactic treatment options in preventing migraine attacks [8,9]. In recent years, different peptides related to CGRP in the calcitonin peptide family have been proposed to have a role in the pathophysiology of migraine [1,10].

AM entered the literature in the early 1990s [11]. Later studies revealed that it is secreted from many different tissues and is an essential peptide in many areas including angiogenesis [12,13]. Some studies have also suggested a possible role of AM in pain processing [1,14]. Contrary to a previous study [15], a recent study reported that AM infusion can initiate an attack more often than a placebo in migraine patients [16].

Despite the developing technology and intensive research in recent years, migraine pathophysiology remains unclear. There is insufficient information about the pathophysiology of the attack. The pain pathways and responses of people with migraine to sensory stimuli may differ in both ictal and interictal periods [17,18]. Additionally, the pathophysiological mechanisms that occur in provoked attacks may not be similar to unprovoked attacks [19].

In this study, we measured and compared serum CGRP and AM levels simultaneously during unprovoked attacks and in the interictal period of migraine patients. The findings of the migraine group were also evaluated according to the results of the control group. Additionally, we evaluated whether serum CGRP and AM levels showed a relationship with the frequency of headache attacks in the last three months, the severity of headaches, provoking factors, and accompanying clinical findings.

## Materials and methods

Migraine patients admitted to Sanko University and Dr. Ersin Arslan Education and Research Hospital Neurology outpatient clinics aged between 18 and 50 years were asked whether they agreed to apply to the neurology outpatient clinics two more times to either of the hospitals for blood tests; once during a migraine attack without taking any medication, and once during the interictal period. Patients who consented to the study protocol were included in the study. The diagnosis of migraine with or without aura was made according to the International Headache Society diagnostic criteria [20]. The control group included individuals who did not have any primary headache disorders in the same age group. Hypertension, renal dysfunction, endocrinological or rheumatological disease, and signs of active infections were the exclusion criteria for both the patient and control groups.

Sociodemographic characteristics; drugs used in the last month; severity, duration, and frequency of headache attacks; lost productive time in work and/or household work and social life due to headache in the last three months; duration of attacks; provoking factors; and accompanying findings were recorded.

In this study, 10-15 mL of venous blood samples were collected from the cubital vein during ictal and interictal periods of migraine patients and at any time from the control group. The blood samples during the attacks were taken before the patient took any medication for the present attack.

Blood was drawn from all subjects participating in the study in outpatient clinics. Because the first blood was drawn during an attack in the patient group, we could not designate a constant time for drawing blood. and blood samples were taken at any time when the patients presented with an attack. However, all blood samples were obtained during working hours in the daytime and stored at -80°C until the laboratory evaluation after separating the serum.

Ictal and interictal serum CGRP and AM levels were assessed to determine significant differences and were compared with the results of 25 healthy controls. Ictal serum CGRP and AM levels were also evaluated according to the clinical features to determine whether they had a relationship with the frequency of headache attacks in the last three months, the severity of headaches, provoking factors, and accompanying features of migraine attacks.

AM and CGRP measurements

The blood samples of the included cases were taken into yellow-capped tubes and centrifuged at 3,500 rpm for five minutes. Subsequently, their serum was aliquoted, and stored at -80 °C in Sanko University Central Laboratory Facility until the day of the study. AM and CGRP concentrations were determined using enzyme-linked immunosorbent assay kits (Elabscience E-EL-H0275, E-EL-H0619). Results are presented as pg/mL.

Ethical considerations

Informed consent was obtained from all participants included in this study. All procedures performed in this study involving human participants were in accordance with the ethical standards of the institutional and national research committee and with the 1964 Declaration of Helsinki. Approval was obtained from the Ethics Committee of Sanko University Faculty of Medicine (Date: 17/09/2020, Session number: 2019/13, Decision number: 11).

Statistical analysis

The normality of the distribution was tested using the Shapiro-Wilk test. Student’s t-test and Mann-Whitney U-test were used to compare numerical variables between groups. Paired-sample t-test and Wilcoxon test were applied for intragroup group comparisons. The chi-square test was conducted to investigate the relationship between categorical variables. Statistical analysis was performed using SPSS for Windows version 24.0 (IBM Corp., Armonk, NY, USA). P-values <0.05 were considered statistically significant.

## Results

A total of 30 migraine patients (26 females and four males) between the ages of 18-41 years and 25 control cases (21 females and four males) between the ages of 22-40 years were included in this study (Table 1).

**Table 1:** Age, gender, serum CGRP, and AM levels of the patient and control groups. M/F: male/female; n: number; min: minimum; max: maximum; CGRP: calcitonin gene-related peptide; AM: adrenomedullin

	Patient (n = 30)	Control (n = 25)	P-value
Gender (M/F)	4/26	4/21	0.780
Age (min-max), years	18–41	22–40	0.628
Median (25%–75%)	25 (23–32)	25 (23–28)	

Overall, 40% of patients had a headache for more than five years, with nearly two-thirds reporting lost productive time due to headaches more than one day per month. Moreover, 80% of the patients had at least one attack per month, and the duration of the attacks was less than 24 hours in 53.3% of the patients. Headaches were generally defined as severe/very severe in more than 80% of the patients.

The most frequently described provoking factors were psychological stress, changes in sleep patterns, and exhaustion (86.7%, 83.3%, and 70%, respectively) (Table 2). Distraction, mood changes, and sweating/chill feeling were the most common findings accompanying headache during attacks (83.3%, 80%, and 70%, respectively) (Table 2).

**Table 2:** Clinical characteristics associated with attacks.

	n (%)
Lost productive time due to headaches	
No	2 (6.7)
More than one day a month	12 (73.3)
Less than one day per month	6 (20.0)
Duration of attacks	
4–24 hours	16 (53.3)
25–72 hours	12 (40.0)
Longer	2 (6.7)
Frequency of headache attacks in the last three months	
At least once a week	13 (43.3)
Less than once a week	12 (56.7)
Provoking factors	
Psychological stress	26 (86.7)
Changes in sleep patterns	25 (83.3)
Physical stress	21 (70.0)
Findings accompanying headaches	
Distractibility	25 (83.3)
Mood change	24 (80.0)
Sweating/chills	21 (70.0)

The serum CGRP and AM levels of the migraine group in both ictal and interictal periods and the control group were similar (Table 3). No significant relationship was determined between the clinical features of migraine attacks and CGRP and/or AM levels in the ictal or interictal periods.

**Table 3:** Serum CGRP and AM levels of the patient in ictal and interictal periods and control groups. Significant at 0.05 level; Mann-Whitney U test for between groups; Wilcoxon test for intragroup comparisons. AM: adrenomedullin; CGRP: calcitonin gene-related peptide

	Patient (n = 30)	Control (n = 25)	P-value
CGRP during an attack (pg/mL)	2.93 (2.45-3.90)	3.03 (2.48-3.80)	0.987
Attack-free CGRP (pg/mL)	3.25 (2.85-4.67)	3.03 (2.48-3.80)	0.139
CGRP within groups (pg/mL)	P = 0.054		
AM during an attack (pg/mL)	15.80 (11.91-21.43)	13.36 (10.84-17.18)	0.230
Attack-free AM (pg/mL)	15.85 (12.25-19.29)	13.36 (10.84-17.18)	0.295
AM within groups (pg/mL)	P = 0.558		

## Discussion

In this study, we found that serum CGRP levels were similar in ictal and interictal periods in migraine patients without a significant difference from the control group. Due to its receptors’ anatomical localization and physiological functions, a critical role in migraine attacks has been suggested for CGRP. However, in studies conducted to date, serum CGRP levels in the ictal period in migraine patients have been mostly inconsistent.

About 30 years ago, increased CGRP levels in blood samples taken from the jugular vein in migraine attacks were reported that supported the presumption that CGRP may have a crucial role in migraine [21]. Therefore, some agents that block CGRP and its receptors that may potentially reduce migraine attacks have been investigated [5-7]. Over time, clinical trials have been conducted with various agents, and CGRP blockade in migraine patients has been found to be statistically more beneficial than placebo in phase 3 studies.

However, some recent meta-analyses revealed that the power of CGRP blockade to prevent migraine attacks may not be higher than the current prophylactic treatment options [8,9]. Additionally, subsequent studies investigating serum CGRP levels during migraine attacks over the years have reported conflicting results [22-24]. Although there are some methodological differences from the previous studies [21], a recent study performed with the blood samples from the jugular vein reported that ictal serum CGRP levels were not different from that of the interictal period and controls [22]. Our results supported studies that found ictal serum CGRP levels similar to the interictal period and the controls. We wish to emphasize that we excluded chronic migraine patients from this study. CGRP has an essential role in pain processing that is more significant in chronic pain states. The studies that reported high serum CGRP levels did not exclude chronic migraine patients in the study design [25] which may have an influence on the interpretation of results.

In recent years, the view that different peptides related to CGRP may play a role in the pathophysiology of migraine has emerged [1,26]. Amylin has been reported as a candidate molecule in this respect [10,27]. Another peptide in the calcitonin family is AM. CLR is the common receptor that CGRP and AM use [1]. RAMPs determine which ligand the CLR will bind to either CGRP or AM.

AM and related structures are detected in the trigeminovascular structure and in the peripheral cells, such as satellite glia, and in the dorsal root ganglia, as well as in the spinal cord [1,28]. It may have a role in pain processing [1,14]. Contrary to a previous study [15], a recent study performed among migraine patients revealed that AM infusion initiates migraine attacks more than placebo [16]. In this study, ictal serum AM levels did not show a significant difference from the interictal period or from the control group. Our study results may be admitted as one of the first in the literature. Because we do not have sufficient data about the ictal or interictal serum AM levels in migraine due to a lack of previous studies, it is not easy to interpret our results.

Although it is determined by the diagnostic criteria, the characteristics of the headache, the presence and severity of nausea and vomiting, and light and sound sensitivity during attacks vary from patient to patient and even from attack to attack in the same patient. The same coloration is similar regarding accompanying findings, such as gastrointestinal motility, mood changes, and facial flushing. We do not have data on whether accompanying findings or headache-provoking factors are associated with CGRP or AM. This study demonstrated no correlation between serum AM and/or CGRP levels with the frequency, severity, provoking factors, and accompanying clinical findings of headaches in the last three months.

The intricate interactions between peptides of the calcitonin family and their receptors complicate the evaluation of the pharmacokinetic consequences of the drugs with either agonist or antagonist effects [1]. In other words, the levels of other peptides using the same receptors or changes in their binding properties may increase or decrease the effect of CGRP. An experimental study in microvascular endothelial cell culture investigated how endothelial endoCL receptors would behave when AM, CGRP, and their antagonists were added to the medium [29]. In this study, it was shown that endoCL internalization, which is formed by the addition of AM to the medium, can be blocked by both AM and CGRP antagonists (AM22-52 and CGRP8-37), and the receptor desensitized by binding with AM also desensitizes against CGRP [29]. Although the addition of CGRP to the medium did not cause receptor internalization, it desensitized the receptor to both CGRP and AM [29]. In summary, binding one of the two peptides in the medium (AM or CGRP) to the endogenous CL desensitized the receptor to both peptides. It is not yet known whether such an interaction exists in migraine. Investigating whether there are interactions of these peptides in the natural processes of migraine may add to our knowledge.

So far, none of the hypotheses put forward could fully explain all the clinical features seen in migraine. We have very limited information about the interictal processes that lead to attacks in migraine. Furthermore, the factors that lead to an attack may not be the same as those that occur during the ictal period. It can be predicted that a follow-up study including more than one evaluation between and during attacks, including other laboratory evaluations such as functional brain imaging in migraine cohorts that are well homogenized according to migraine characteristics, will provide more information than a cross-sectional assessment.

Chronic migraine patients were not included in this study. However, patients with migraine with and without aura were included in the study and the cohort was relatively small. These are the main limitations of this study. Some previous reports have revealed that peripheral structures play a role in the onset of headaches in migraine [30]. Besides, migraine drugs target peripheral structures mostly. Consequently, the changes that occur during migraine attacks may also be reflected in the peripheral blood. However, these are only assumptions, and evaluation of blood obtained from more central veins might provide additional data. Therefore measuring serum CGRP and AM levels from peripheral blood may be another limitation of the study.

## Conclusions

According to the results of this study, serum AM and CGRP levels in migraine patients in the interictal period and unprovoked migraine attacks are similar to controls. These results do not indicate that these molecules do not have a role in migraine pathophysiology or do not have an effect on peripheral structures associated with pain. However, it may highlight a need for studies to investigate mediators that may have a role in the pathophysiology of migraine and are likely to be present in the circulation with different methods than measuring blood levels.
